# Challenges of defining a non-inferiority margin: a case study of non-inferiority randomized controlled trials of oral anti-thrombolytic agents for prophylaxis of venous thromboembolic events after orthopedic surgery

**DOI:** 10.1186/1745-6215-12-S1-A32

**Published:** 2011-12-13

**Authors:** Grace Wangge, Olaf H Klungel, Kit CB Roes, Antonius de Boer, Arno W Hoes, Mirjam J Knol

**Affiliations:** 1 Department of Pharmacoepidemiology & Clinical Pharmacology, Utrecht Institute for Pharmaceutical Sciences (UIPS), University of Utrecht, The Netherlands; 2 Julius Center for Health Sciences and Primary Care, University Medical Center Utrecht, The Netherlands

## Objective

To identify problems and difficulties in determining a non-inferiority (NI) margin using the case of NI randomized controlled trials (RCTs) of oral anti-thrombolytic agents for prophylaxis of venous thromboembolic events (VTE) after orthopedic surgery.

## Methods

We searched in Pubmed and Cochrane-central-register-for-controlled-trials for all NI RCTs of direct thrombin inhibitors (DTI) and direct inhibitors of factor Xa (DXAI) for prophylaxis of VTE. All NI trials had enoxaparin as their active comparator. Using the draft FDA guidelines for NI trials, we determined an NI margin, referred to as the reference NI margin, based on all published placebo-controlled trials on enoxaparin for the same indication, identified in PubMed and Cochrane-central-register-for-controlled-trials. We used preserved-effects of 50% and 67% to calculate the reference NI margin.

## Results

We identified 12 NI trials and 4 placebo-controlled trials of enoxaparin from our searches. All NI trials studied oral drugs. Trials in DTI used the risk difference (RD) to define their NI margin, and it ranged from 0.02 to 0.092. Trials in DXAI used the RD (ranging from 0.035 to 0.056) or risk ratio (RR) (1.25) or both to define their NI margin. Furthermore, the NI margins using the RD were stricter than the 50% preserved-effects reference NI margin ((0.02 to 0.092) vs. 0.115). The NI margins in the trials using the RR were stricter than the 50 and 67% preserved-effects reference NI margin (1.25 vs. 1.46 and 1.28). In one trial, the test drug might have been concluded as non-inferior to enoxaparin if the 50% preserved-effects reference NI margin of RR were used.

## Conclusions

Although a same comparator was used, a large variation in NI margins among NI RCTs of oral anti-thrombolytic agents for prophylaxis of VTE after orthopedic surgery exists. Using different NI margins could lead to different conclusions of the drug's efficacy. Challenges that became apparent during determination of an NI margin were 1) missing unpublished results of placebo-controlled trials, 2) how similar should placebo-controlled trials and NI trials be to maintain the constancy assumption, 3) whether fixed or random effects analysis should be used in the meta-analysis, 4) whether to calculate the NI margin on an absolute or relative scale, 5) which preserved-effects to use, and 6) whether further clinical judgment is needed.

